# Risk Factors for Canine Osteoarthritis and Its Predisposing Arthropathies: A Systematic Review

**DOI:** 10.3389/fvets.2020.00220

**Published:** 2020-04-28

**Authors:** Katharine L. Anderson, Helen Zulch, Dan G. O'Neill, Richard L. Meeson, Lisa M. Collins

**Affiliations:** ^1^School of Life Sciences, University of Lincoln, Lincoln, United Kingdom; ^2^Dogs Trust, London, United Kingdom; ^3^Royal Veterinary College, London, United Kingdom; ^4^Faculty of Biological Sciences, University of Leeds, Leeds, United Kingdom

**Keywords:** canine, dog, degenerative joint disease, osteoarthritis, risk factor, systematic review

## Abstract

Osteoarthritis is a common clinical and pathological end-point from a range of joint disorders, that ultimately lead to structural and functional decline of the joint with associated lameness and pain. Increasing understanding of the risk factors associated with osteoarthritis will assist in addressing the significant threat it poses to the welfare of the dog population and implementing preventive measures. Presented here, is the first comprehensive systematic review and evaluation of the literature reporting risk factors for canine osteoarthritis. This paper aimed to systematically collate, review and critically evaluate the published literature on risk factors for canine osteoarthritis and its predisposing conditions such as developmental joint dysplasias, cruciate ligament degeneration, and patellar luxation. Peer-reviewed publications were systematically searched for both osteoarthritis and predisposing arthropathies on Web of Science and PubMed following PRISMA (2009) guidelines, using pre-specified combinations of keywords. Sixty-two papers met the inclusion criteria and were evaluated and graded on reporting quality. Identified risk factors included both modifiable factors (neuter status and body weight) for which intervention can potentially affect the risk of occurrence of osteoarthritis, and unmodifiable factors (sex, breed, and age) which can be used to identify individuals most “at risk.” Osteoarthritis in dogs frequently develops from predisposing arthropathies, and therefore risk factors for these are also important to consider. Papers evaluated in this study were rated as medium to high-quality; gap analysis of the literature suggests there would be significant benefit from additional research into the interactions between and relative weighting of risk factors. There are a number of examples where research outcomes are conflicting such as age and sex; and further investigation into these factors would be beneficial to attain greater understanding of the nature of these risks. Comprehensively collating the published risk factors for osteoarthritis and its predisposing conditions offers opportunities to identify possible means for control and reduction within the population through preventative methods and control strategies. These factors are highlighted here, as well as current literature gaps where further research is warranted, to aid future research direction.

## Introduction

Osteoarthritis, a common pain-causing condition of synovial joints, affects millions of human and non-human animals worldwide (1). Osteoarthritis—otherwise referred to as osteoarthrosis or degenerative joint disease—is a disease of the entire joint organ, including all its associated tissues, but is most frequently associated with the loss and dysfunction of articular cartilage (2). The etiology of osteoarthritis is complex and the specific pathways that lead to its development remain uncertain (3). In humans, reported risk factors for development of osteoarthritis are manifold with both systemic and local causes, linked to factors including: genetics, age, sex, obesity, previous joint trauma, and underlying diseases such as cruciate ligament rupture and osteochondritis dissecans (1). Although osteoarthritis has been reported in a wide range of non-human species, the prevalence of the condition in many of these species remains largely unexplored and as such underreported (4).

With an estimated 9 million pet dogs owned in the UK (5), and 63.4 million households in the US owning a dog (6), the disease burden of osteoarthritis to dogs worldwide is considerable and poses a significant threat to canine welfare. Osteoarthritis prevalence in North America is reported at 20% of all dogs over 1 year of age based on data collected from 200 veterinarians (7). Recent prevalence estimates (likely heavily underestimated due to the nature of reporting methodology) for osteoarthritis in the UK dog population vary widely, from 2.5 and 6.6% of dogs of any age and breed attending primary-care practices [estimates respectively from (8, 9), and up to 20% of dogs over 1 year of age (10)]. In addition to the welfare impact for dogs, canine osteoarthritis is also a major issue worldwide for veterinarians, owners and breeders. Canine osteoarthritis can particularly impact an owner's welfare, with treatment plans having considerable financial costs. For example $1.32bn was spent on cruciate ligament ailments alone in dogs in the US in 2003 (11). There is also the emotional cost to the owner dealing with an animal that is chronically or terminally unwell and/or in chronic pain, which can cause psychological distress and upset known as caregiver burden (12).

Primary osteoarthritis is described as largely idiopathic, but can be associated with several risk factors including aging and obesity (13). Secondary osteoarthritis, where underlying disease processes or injuries play a role in the development of osteoarthritis, is believed to be the most common form in dogs (14). The pathogenesis of secondary osteoarthritis is considered to have a genetic component exacerbated through aspects of lifestyle that impact body condition, such as diet and exercise (15). Disease processes and pre-existing arthropathies often influence the pathogenesis, for example cranial cruciate ligament disease is a common cause of pelvic limb lameness and can result in osteoarthritis development in breeds of all sizes (16). Joint dysplasia, commonly occurring in the hip or elbow, describes failure of normal joint formation during development and can lead to well-recognized and described joint conditions which cause pain and lameness in their own right, and can progress to osteoarthritis (17). Consequently, it is important to understand the risk factors for these complex diseases when considering the epidemiology of canine osteoarthritis.

A critical evaluation of the existing published evidence on risk factors for osteoarthritis and its predisposing conditions is required in order to assess what is known and where the key gaps in knowledge remain. Here, a comprehensive systematic review and evaluation of literature reporting risk factors for canine osteoarthritis is presented. Within this review, the published risk factors associated with the development of both osteoarthritis and predisposing conditions are highlighted, the reporting quality of current evidence is evaluated and recommendations for future research based on existing findings and gaps in knowledge are discussed.

## Materials and Methods

### Literature Search

#### Stage 1—Identification

**Figure 1:**
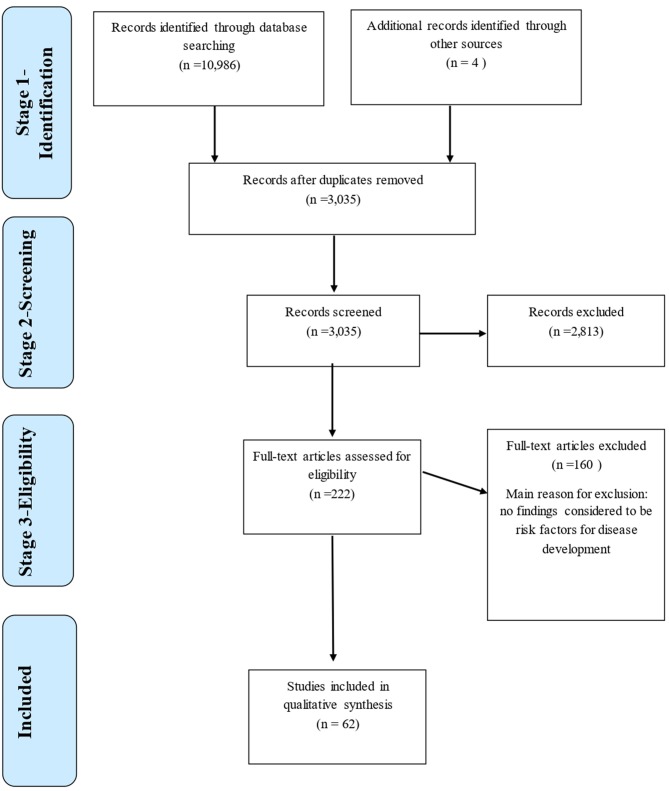
Flowchart adapted from PRISMA Guidelines, 2009 (18) of the literature search strategy used to identify articles with information on risk factors for canine osteoarthritis and its predisposing conditions, with 62 studies retained for further quality evaluation.

The peer-reviewed literature was systematically searched for papers which may have included risk factors associated with canine osteoarthritis and its predisposing conditions, using the approach outlined by the PRISMA (2009) guidelines [(18); Figure 1]. The online databases Web of Science (WoS) and PubMed were used to generate broad searches using key topic words within logical sequences incorporating Boolean operators (“AND” and “OR”) to ensure papers included (within any part of the paper), the keywords of interest (Table 1). All identified papers from each search were stored in a Microsoft Excel database. Data stored included author names, year of publication, paper title, journal title, issue, volume, and page numbers. Literature searches were conducted during March 2019.

**Table 1:** Search terms used for systematic review literature search (156 combinations in total) conducted on Web of Science and PubMed to obtain literature surrounding risk factors for canine osteoarthritis and its predisposing conditions.

**Species**	**AND**	**Disease**	**AND**	**Keywords**
Dog		Degenerative joint disease		Risk Factor^*^
OR		OR		OR
Canine		Osteoarth^*^		Predictor^*^
		OR		OR
		Dysplas^*^		Susceptibility
		OR		OR
		Dislocat^*^		Cause
		OR		OR
		Joint fracture		Prevalence
		OR		OR
		Ununited anconeal process		Incidence
		OR		
		Luxat^*^		
		OR		
		Cruciate ligament		
		OR		
		Developmental elbow disease		
		OR		
		Fragmented coronoid process		
		OR		
		Osteochondrosis		
		OR		
		Osteochondritis dissecans		

* *Asterix used as wildcard symbol allowing for variations and spellings of words that start with the same letters*.

#### Stage 2—Screening

Papers identified during Stage 1 were carried forward to Stage 2. They were initially sorted by title by one researcher (KA); the title had to include reference to dog/canine and osteoarthritis (or a synonym) or an associated disorder (listed in Table 1). Included papers had to evaluate at least one risk factor as suggested by the inclusion of words in the title such as but not limited to “risk factor,” “prevalence,” “predictors,” or “susceptibility.” Screening stage lists (300 randomly selected papers per reviewer) were independently evaluated by two additional reviewers (1 HZ, 2. LC). Inter-observer reliability (the degree of consistency in selecting the papers between all three researchers) was calculated using percentage of agreement. In the case where there was disagreement, for example where human error occurred, the list was re-reviewed (by KA) and papers were included or discarded upon second review of the title.

#### Stage 3—Eligibility

Papers retained from Stage 2 moved to Stage 3, which involved firstly checking the abstract for relevance to the inclusion criteria. Papers that were retained through the abstract checks were then read in full and either retained or excluded based on their match to the inclusion criteria described below.

#### Final Corpus

The reference lists of all papers included in the final corpus were checked and citations not already captured in the literature search to date were screened from Stage 2 onwards. Papers within the final corpus were categorized based on the primary disorder of focus. Most papers investigated a single disorder and area of risk; where multiple disorders were reported, each paper was categorized based on which disorder featured most prominently.

### Inclusion Criteria

Papers published in peer-reviewed journals were included in the search with no timeframe filter. Included papers were either written in or translated into the English language and no filters were included on country of origin.

Papers were included in the final corpus following the title, abstract and full text check only if they were:

- Peer reviewed papers in the English language including the topic of canine/dog osteoarthritis (and synonyms) or a predisposing condition. Whilst papers that reported on other species in addition to dogs were included, only the results related to dogs were included within this review.
- Reporting primary research (literature reviews were excluded);
- Reporting research that applied statistical testing to demonstrate increased risk of disease or demonstrated variation in susceptibility to develop or be diagnosed with osteoarthritis or a predisposing condition, such as (but not limited to) genetic or biomarker studies (due to diverse methodologies used in epidemiologic studies, no types of study design were excluded);
- Inclusion of dogs that had been reported to have osteoarthritis (or synonym) or one of its predisposing conditions, apart from in the case of “healthy” control cases.

### Reporting Quality Evaluation (QE)

Eligible full text papers were subject to reporting quality evaluation (QE). The quality appraisal tool was created during this study based on adaptations from the Critical Appraisal Skills Program checklist (19). The tool was adapted to assess reporting paper quality by evaluating the reporting of methodology (including risk of bias) and outcomes/results. QE was scored as high (QE-H): 8–10, medium (QE-M): 4–7, or low (QE-L): 0–3 (Table 2).

**Table 2:** Information recorded as evaluation criteria of reporting quality based on recommendations from the Critical Appraisal Skills Program (CASP, UK) (19), used to assess the reporting quality of current published evidence of risk factors for canine osteoarthritis and its predisposing conditions as part of the systematic review.

**Area of evaluation**	**Answer and score awarded**	
	**Yes**	**No/Not stated**
Is there a clear research question, aim or hypothesis and does the study design suitably answer it with appropriate statistical analysis and results stated (values)?	1	0
Was the study period a suitable time frame?	1	0
Is the study design relevant to answer the study question?	1	0
Is the research applicable to the target population?	1	0
Are there any other explanations for the conclusions discussed? (e.g., other confounding variables, result variability due to methods)	1	0
Does the conclusion fit with other studies?	1	0
Does the study provide the full picture so that it is repeatable?	1	0
Was there use of controls?	1	0
Any bias in patient selection?	0	1 (or Y but acknowledged)
Does the research hold any implications (either positive or negative)?	1	0

*The maximum possible score was 10*.

The following details for each paper in the final corpus were recorded in a Microsoft Excel spreadsheet:

- Publishing details: paper title, authors, year of publication, journal name, volume number, and page numbers;
- Study details: disease of focus, study design, statistical analyses (test/s used), overall sample size (total number of dogs included in study including controls where used), and control sample size (number of control dogs where used), whether sample size/power analysis was calculated and reported in the paper;
- Study outcomes: risk factors identified (qualitatively recorded), and the direction and measurement of the risk (whether it increased or decreased likelihood of osteoarthritis development).

## Results

### Study Selection

At Stage 1, 10,986 papers were returned by searches in Web of Science and PubMed and stored in Endnote. Once duplications had been removed, 3,033 papers were in the pre-screened corpus, and were exported to Excel for inclusion screening. Following Stage 2 screening, 479 paper titles were retained; During Stage 3, after abstract checks, 220 papers were retained (Figure 1). After full texts were checked, a total of 57 papers met the inclusion criteria to be included in the Final Corpus. The Final Corpus totaled 62 papers that met the inclusion criteria for review discussing risk factors associated with joint conditions (57 of these from the database search, 4 known separately to the authors, 1 from reference list searches within included papers).

Inter-observer reliability for percentage agreement of papers obtained in the screening stage between the three independent assessors was calculated at 97% across title checks (582/600; 300 papers were randomly selected each and reviewed by the two additional reviewers).

From the Final Corpus of 62 papers, the main disease of focus (i.e., within the paper title or where multiple diseases were discussed, the primary disease of interest) for 20 (32%) papers was hip or elbow dysplasia, 17 (27%) focused on cruciate ligament disease, 16 (26%) on osteoarthritis, 6 (10%) on patellar luxation, and 3 (5%) on osteochondritis dissecans.

### Study Characteristics and Reporting Quality Evaluation

Regarding study design, 31 (50%) studies were retrospective cross-sectional, 16 (26%) were retrospective case-control, 11 (18%) were prospective cohort studies, three (5%) were prospective cross-sectional studies and one (2%) was a retrospective cohort study. The existing literature has a wide timespan with publication dates ranging from 1972 to 2019 (47 years). The majority of papers (53%) were published since 2009, 22 since 2014. For papers published since 2014, the disease most frequently in focus was cruciate ligament disease (eight papers; 36%), followed by hip dysplasia (five papers; 23%), patellar luxation (five papers; 23%), osteoarthritis (three papers; 13%), and osteochondritis dissecans (one paper; 5%) (see Table 3 for study design for each individual paper).

**Table 3:** Study reporting quality evaluation results and information recorded for the 62 studies that met the systematic review inclusion criteria for canine osteoarthritis and predisposing conditions risk factors.

**References**	**Risk factor paper findings**	**Direction of risk**	**Reporting quality evaluation (QE) category and score**	**Type of study**	**Overall sample size**	**Control sample size**	**Sample size calculated**
**Cruciate ligament literature evaluation**							
Adams et al. (20)	Females	Increased (OR 2 compared to males)	H-9	R-CC	1,368	1,179	N
	Rottweiler breed	Increased (OR 5 compared to crossbreeds)					
	Obesity	Increased (OR 3.8 compared to healthy weight)					
	Younger dogs	Decreased (OR 0.2 compared to dogs >8)					
Baird et al. (21)	Regions on Chr 3 and 33 (most significant)	Decreased (OR 0.1–0.2)	M-7	R-CC	749	456	N
	Regions on Chr 1 (most significant)	Increased (OR 5.96)					
Baird et al. (22)	Collagen genes significantly associated	Increased	M-7	R-CC	271	172	N
Baker et al. (23)	Multiple genetic loci (~172) contribution	Increased	M-7	R-CS	237	139	Y
	Heritability 0.48						
Baker et al. (24)	Significant loci on ROR2 (Cartilage and bone development)	Increased	M-7	R-CS	222	69	N
	Significant loci on DOCK2 gene (immune cell migration)	Increased					
Clements et al. (25)	Neutered	Increased	H-8	R-CC	17	12	N
	COL5A1 and RPL13A upregulated in	Increased					
	14 genes upregulated in rupture	Increased					
	2 genes down regulated in rupture	Increased					
Duval et al. (26)	Large breeds (9 predisposed)	Increased (OR range 2.15–15.33)	H-10	R-CC	1,005	804	N
	Neutered	Increased					
	Greater body weight	Increased					
Grierson et al. (27)	Rottweilers Golden Retriever	Increased (OR 1.89) Decreased (OR 0.36)	H-9	R-CS	511	N/A	N
	Males	Increased (OR 1.72)					
	Overweight	Increased (OR 1.77)					
Guenego et al. (28)	High tibial anatomical-mechanical axis angle	Increased	H-9	R-CS/CC	274	72	N
Inauen et al. (29)	Lower tibial tuberosity width	Decreased	H-8	R-CS	219	73	N
	Greater body weight	Increased					
	Larger proximal tibial tuberosity angle	Increased					
	Younger	Decreased					
Morris and Lippowitz (30)	Larger tibial plateau angle	Increased	H-8	P-C	87	31	N
Necas et al. (31)	Breeds: Am. Staff terrier, Rottweiler,	Increased	H-9	R-CS	183	N/A	N
	Chow Chow, St Bernard, Bullmastiff	Increased					
	German shorthaired pointer, Boxer	Increased					
	German Shepherds	Decreased					
Pecin et al. (32)	5–8 years	Increased	M-7	R-CS	117	N/A	N
	Mixed breeds and Labradors	Increased					
Taylor-Brown et al. (16)	Neutered females	Increased	H-9	R-CC	2,828	1,875	Y
	>3 years	Increased (OR 2.1)					
	Rottweiler, West Highland Terrier, Golden Retrievers, Yorkshire Terriers, and Staffordshire Bull Terriers	Increased (OR 5.4, 2.5, 1.9, 1.8, respectively)					
	Cocker Spaniels	Decreased (0.4)					
	Increasing body weight	Increased (OR 3.4)					
	Insured	Increased (OR 4.0)					
Townsend et al. (33)	Steep medial tibial plateau midsagittal radius of curvature (m-TPr) angle	Increased	M-7	R-CS	18	18	N
Whitehair et al. (34)	7–10 years	Increased	H-10	R-CC	602,317	591,548	N
	Neutered	Increased					
	Females	Increased					
	Rottweiler, Newfoundland, Staff terrier	Increased					
	Old English Sheepdogs, Basset Hounds, and Dachshunds	Decreased					
	Greater body weight	Increased (>22 kg)					
Wilke et al. (35)	86 markers associated with CCLR traits	Increased	M-6	R-CS	90	N/A	N
	4 associated markers on chr 3, 5, 13, and 24	Increased					
**Dysplasia literature evaluation**							
Beuing et al. (36)	Males	Increased	H-8	R-CS	2,114	N/A	N
	Heritability estimate 0.28	Increased					
Cardinet et al. (37)	Low Pelvic muscle mass index	Increased	H−8	P-C	82	N/A	N
Choi et al. (38)	High distraction index	Increased	M-5	R-CS	87	N/A	N
	Greater weight	Increased					
	Dogs kept indoors through growth	Increased					
Clements et al. (39)	5 SNPs associated with risk	Increased	M-5	R-CC	647	438	N
	5 SNPs associated with protection	Decreased					
	8 haplotypes as risk (5) or protectors (3)	Increased and Decreased					
Coopman et al. (40)	German Shepherd dog, Golden and Labrador retriever and Bernese Mountain dog (Hip)	Increased (prevalence)	M-6	R-CS	7,506	N/A	N
	Rottweilers, Newfoundland, and Sharpei (elbow)	Increased (prevalence)					
Hou et al. (41)	Boykin Spaniel and St Bernard (Hip)	Increased (Incidence)	H−8	R-CS	895,864	N/A	N
	Siberian Husky and Afghan Hound (Hip)	Decreased (Incidence)					
	Rottweiler (elbow)	Increased (Incidence)					
	Rhodesian Ridgeback (Elbow)	Decreased (Incidence)					
	Males (elbow)	Increased					
	3–5 years old	Increased					
Kealy et al. (42)	Non-limited feeding	Increased	M-7	P-C	48	N/A	N
Krontveit et al. (43)	Born Spring and Summer	Decreased	H-8	P-C	501	N/A	N
	Urban/suburban home (breeder home)	Increased					
	Exercise on soft ground, daily stair use	Increased					
	Off leash exercise (from 0 to 3 months)	Decreased					
Lavrijsen et al. (44)	Bullmastiff, Boxer, and Italian Corso dog most prevalent	Increased (prevalence)	H-9	R-CS	35,046	N/A	N
	Golden Retrievers—Female	Increased (prevalence)					
	Labrador Retriever—Males	Increased (prevalence)					
Lavrijsen et al. (45)	Associated regions on chr 8	Increased	H-9	R-CC	122	NS	N
	Candidate genes LAMA2, LRR1, and COL6A3 (disruption in etiology of hip)	Increased					
Leppanen et al. (46)	Born spring and summer	Decreased	H-8	P-CS	10,335	N/A	N
	Older dogs	Increased					
Loder and Todhunter (47)	Females	Increased (OR 1.05)	H-8	R-CS	921,046	N/A	N
	Born in spring and winter	Increased (OR 1.14 and 1.13)					
	Working dogs	Increased (OR 1.88)					
Oberbauer et al. (48)	Increasing age	Increased	H-9	R-CS	1,331,981	N/A	N
	Heritability 0.57	Increased					
Priester and Mulvihill (49)	Large and giant breeds	Increased (Relative risk 3.6 and 10.2)	H-9	R-CS	1,193	N/A	N
	Small and medium breeds	Decreased (Relative risk 0.2)					
Sallander et al. (50)	Exercise by running after balls/sticks	Increased (OR 2.4)	M-6	R-CC	292	NS	N
	High fat intake/energy from fat	Increased					
	Overfeeding/ High body weight	Increased					
Todhunter et al. (51)	HHIP, DACT2, and WIF1 expression	Decreased	M-6	R-CC	32	8	N
	SPON 1, FBN2, EMILIN3, ACAN, IGF1, CILP2, COL11A1, COL8A1, HAPLN, PLA2F, TNFRSF, TMEM, IGFBP expression	Increased					
Torres de la riva et al. (26)	Early neutered males	Increased	M-7	R-C	1,518	N/A	N
Witsberger et al. (52)	Neutered males	Increased (OR 1.21)	H-8	R-CS	1,243,681	N/A	N
	2 months−1 year and 1–4 years	Increased (OR 1.22 and 1.48)					
	Large and Giant breeds	Increased					
Wood and Lakhani (53)	Born July to October	Decreased	M-7	R-CS	9,657	N/A	N
	Parents with high hip scores (parental genetic effect)	Increased					
Worth et al. (54)	Born Autumn (March and April, New Zealand)	Decreased	H-9	R-CS	5,722	N/A	N
**Osteoarthritis literature evaluation**							
Anderson et al. (9)	Rottweiler, Dogue de Bordeaux, and Old English Sheepdogs	Increased (OR 3.1, 2.8, and 2.8)	H-8	R-CS and CC	455,557	451,361	Y
	Insured dogs, Neutered dogs	Increased (OR 2.02, 1.8)					
	Increasing age (>3 years) and high body weight	Increased (OR 3.55–53.89 and 2.29)					
Andrysikova et al. (55)	High levels of GAGs	Increase	H-8	R-CC	36	5	N
	Higher GAGs in obese dogs	Increase					
Grondalen and Lingaas (56)	Males	Increased	M-6	P-CS	2,046	N/A	N
	Dogs with at least one parent with osteoarthritis	Increased (Relative risk 1–6)					
Hays et al. (57)	Males (increased hip score and risk of osteoarthritis)	Increased	M-7	P-CS	137	N/A	N
	Additive inheritance						
Hegemann et al. (58)	Synovial 5D4 and TIMP-1 increased (ACLR)	Increased	H-8	R-CC	133	30	N
	Higher serum 5D4 and 10-fold lower serum TIMP-1 levels (FPC)	Increased					
	Synovial 5D4 and TIMP-1 were upregulated in dogs (patella luxation)	Increased					
Kealy et al. (59)	Non-restricted feeding	Increased	H-8	P-C	48	N/A	N
	Greater norberg angle and early joint laxity	Increased					
Kealy et al. (60)	Higher body weight	Increased	H-8	P-C	48	N/A	N
	Non-restricted feeding	Increased					
Maccoux et al. (61)	IL-1b expression in synovial fluid and fat pad	Increased	M-7	R-CC	13	5	N
	IL-6 expression in synovial membrane	Increased					
	Synovial membrane IL-8 expression	Decreased					
	IL-10 gene expression in synovial membrane	Increased					
Mayhew et al. (62)	Caudolateral curvilinear osteophytes present	Increased (7.9 times)	M-7	R-CS	25,968	N/A	N
	High distraction index	Increased					
Powers et al. (63)	Caudolateral curvilinear osteophytes present	Increased (3.7 times)	M-7	P-C	48	N/A	N
	Non-restricted feeding	Increased					
Ramirez-Flores et al. (64)	Females	Increased	M-6	P-C	44	N/A	N
	Body weight >10 kg	Increased					
Runge et al. (65)	Non-restricted feeding	Increased	M-7	P-C	48	N/A	N
Runge et al. (66)	High distraction index	Increased (OR by breed)	H-8	R-CS	4,349	N/A	N
	Higher weight	Increased					
	Older dogs	Increased					
Smith et al. (15)	High distraction index	Increased	H-9	R-CS	15,742	N/A	N
	Weight	Increased					
	German shepherd dogs	Increased (4.95 times)					
	Increasing age	Increased					
Smith et al. (67)	Non-restricted feeding	Increased	H-8	P-C	48	N/A	N
Szabo et al. (68)	Circumferential femoral head osteophytes present	Increased	M-7	P-C	48	N/A	N
**Osteochondritis dissecans literature evaluation**							
Guthrie and Pidduck (69)	Males	Increased	M-6	R-CS	46	N/A	N
	Multifactorial mode of inheritance						
	Higher heritability in males	Increased					
Ohlerth et al. (70)	Osteophyte formation	Increased	H-8	R-CS	351	N/A	H
Slater et al. (71)	Drinking well-water	Increased	H-10	R-CC	91	60	N
	Playing with other dogs daily	Increased					
	Feeding specialty dry food	Decreased					
	High dietary calcium	Increased					
**Patella luxation literature evaluation**							
Bound et al. (72)	Small Breeds most prevalent	Increased	H-10	R-CS and CC	155	42	Y
Maeda et al. (73)	Toy Poodles, Pomeranian, Yorkshire Terriers, and Shibas	Increased	M-7	R-CS	2,048	N/A	N
	Genetic- higher risk if littermate has PL	Increased (16.2-fold)					
Nilsson et al. (74)	Heritibality: 0.25 (Chihuahua) 0.21 (Bichon Frise)	Increased	M-6	R-CS	3,095	N/A	N
O'Neill et al. (75)	Small Breeds- Pomeranian, Chihuahua, Yorkshire Terrier, and French Bulldog	Increased (OR 6.5; 5.9; 5.5 and 5.4)	H-9	R-CS	206,482	N/A	Y
	>12 years	Decreased (OR 0.4)					
	Females	Increased (OR 1.3)					
	Neutered	Increased (OR 2.4)					
	Insured	Increased (OR 1.9)					
Srinarang et al. (76)	Significant SNPs in DAG1 gene	Increased	M-7	R-CC	91	30	N
Wangdee et al. (77)	Heritability 0.44	Increased	M-7	R-CS	339	N/A	N
	SNP Chr 13	Increased		+R-CC	96	48	

*C, cohort; CC, case-control; CS, cross sectional; H, high; M, medium; N, no; N/A, not applicable; NS, not stated; OR, odds ratio; P, prospective; R, retrospective; Y, yes*.

Five of the 62 papers (8%) reported a sample size (and accompanying calculation) within their study. All sample sizes and whether a calculation was reported for each paper are included in Table 3. From the Final Corpus, 34 papers (55%) had high reporting quality and 28 (45%) had medium reporting quality (the quality scores for all papers are included within Table 3), with scores ranging between 5 and 10 (the maximum score). The areas where papers most frequently lost points were: they lacked a clear research question, methodology reporting was not detailed enough; and/or there was a risk of bias within the study that was not acknowledged by the authors, for example in sample selection.

### Risk Factor Results

Full results summarizing the risk factor findings for each paper included in this review can be found in Table 3. Across the corpus of papers, 61 (98%) of the papers discussed at least one risk factor that increased the risk (i.e., predisposition toward) of developing a joint disorder, whilst 19 (31%) papers discussed risk factors associated with a decreased risk (i.e., protection against) of joint disorder development. There were six main risk factors (genetics, breed, conformation, age, sex/neuter status, and body weight) reported across the studies, with many studies suggesting joint disease is a multifactorial disorder (Table 3). Other risk factors reported to have an association with disease development included diet/feeding, month of birth and early life factors, exercise levels (particularly when young) and type of exercise, and insurance status (Table 3). The most frequently reported risk factor was genetics (discussed as particular “risk” genes and chromosomal regions, and disease heritability). Twenty-one (34%) of the 62 papers in the Final Corpus reported genetics as a risk factor for osteoarthritis, or a predisposing arthropathy.

#### Direction of Risk

Of the 21 papers that discussed genetics, an increased risk associated with specific genes was reported by 20 of the papers. Genetic factors associated with decreased risk of developing osteoarthritis or a predisposing arthropathy were reported in four papers. Nineteen (30%) papers assessed sex and/or neuter status as a risk, all of which discussed sex (both being male and being female) and neuter status (being neutered) as having an increased risk for joint disorders. Seventeen papers (27%) discussed breed as a risk factor; five of which identified breeds that had a decreased risk, with the remaining describing breeds with increased risk of joint disease. Thirteen (21%) papers assessed body weight all of which should an increase of risk with increasing body weight. Twelve (19%) papers identified age as a risk factor; nine papers found an increased risk of joint disease associated with age (increased risk was recorded in older dogs for osteoarthritis, younger dogs for cruciate ligament disease, and conflictingly both younger and older dogs for hip dysplasia), whilst three papers reported a decreased risk associated with age (decreased risk in younger dogs for cruciate ligament disease, and decreased risk for older dogs for patellar luxation). Finally 10 (16%) papers discussed specific conformational traits associated with either an increased risk of joint disease (9 papers) or a decreased risk (1 paper).

## Discussion

### Reported Risk Factors

The results of this review suggest six key risk factors associated with canine joint diseases. There is currently no weighting applied to risk factors in the current literature, because there are no quantified and validated estimates of their relative influence, and their relative effect on disease development and severity is largely unknown.

#### Genetics

Genetics is seemingly the most influential risk factor, with a large number of papers (21/62) discussing genetics having a significant relationship with specific joint diseases. Whether this reflects the importance of this risk factor for joint diseases, or is resultant of research bias is unclear. Following genome-wide studies, many genes have been identified as being either upregulated or downregulated in affected joints compared to healthy joints, often similar to those genes expressed in human joint diseases (25), and a number of chromosomal regions linked with joint diseases have been identified (Table 3). In many cases, these genes are related to growth and development (21–23, 35, 45).

#### Conformation

Ten studies highlighted that joint disease is affected by conformation, particularly relating to body and leg size, and joint angles required by breed standards, inadvertently making some breeds especially predisposed toward and others significantly protected from development of joint disorders (78, 79). There is however limited evidence for the relationship between conformation and genetics, warranting further research in the area. Traits such as low pelvic muscle mass were reported to increase risk of hip dysplasia (37, 38) and osteoarthritis (59, 62), whilst tibial tuberosity width and angle were associated with increased risk for cruciate ligament disease (28, 29). Breeding to reach desired breed conformational appearances and possible inadvertent co-selection of undesirable musculoskeletal conformations can have detrimental effects on welfare (78). Perhaps as a result of high demand for particular breeds, studies have further recorded constantly increasing inbreeding coefficients increasing susceptibility to inherited disorders such as hip and elbow dysplasia (41). Whilst genetics and conformation are non-modifiable factors at the individual dog level, these could be considered modifiable factors when considering future generations of dogs. Therefore, extreme traits and appearances, as well as breeding programmes and practices need to be addressed in order to reduce the number of conformational defects and inherited disorders, if improvements are to be made to canine welfare. Phenotypic selection of breeding stock based on conformational health as well as reduction in inbreeding coefficients have demonstrated reduced prevalence of joint diseases of the hips and elbows (48) and could prove effective as a preventative measure in certain instances (80). However, some research suggests these schemes may not be as effective as hoped, and therefore further strategies for phenotypic and genetic improvements is needed (46).

#### Breed

Breed was a consistent finding as a common risk factor for joint disease, reported as a risk factor by 17 papers. Certain breeds are discussed as having particular predisposition and risk of joint diseases as a result of both conformation related to breed standards and genetic/heritability components, increasing the likelihood of (but not guaranteeing) the development of joint disease in an individual of that breed compared to other breeds. As a non-modifiable risk factor, this increased risk in susceptibility to joint disease can be used to identify “at risk” individuals by their breed, potentially allowing for earlier diagnoses and treatment. However, it should be noted that in some studies, this increased prevalence may reflect the overall breed popularity and breed prevalence within the dog population [particularly studies that only report prevalence (40, 44) or incidences (41)]. Breeds inclusive of but not limited to Rottweiler, Golden Retriever, and Labrador Retriever were found to have increased risk of cruciate ligament rupture with smaller breeds generally having decreased risk (16, 20, 27, 31, 34). Higher hip and elbow dysplasia prevalence was apparent in larger breeds such as Mastiffs, Boxers, Italian Corso dog, German Shepherds, Golden and Labrador Retrievers, and Bernese Mountain dogs (40, 41, 44, 52) whilst smaller breeds such as Pomeranians, Chihuahua, Yorkshire terrier, and French Bulldog had higher odds of developing patellar luxation compared to crossbreeds (75).

#### Body Weight

Body weight was another important risk factor associated with joint disease development identified here. In some cases, it was unclear whether body weight reflects mainly breed size or body condition. However higher body weight, and thus an increased load on weight-bearing joints (both larger breed dogs, and overweight individuals) was found associated with an increased risk of disease in all papers that it was reported. Overweight dogs were significantly more likely to develop cruciate ligament disorders, with obesity almost quadrupling the risk (odds ratio (OR) 3.8) (20). Having higher body weight related to size or body condition (no OR reported) increased the risk of developing elbow arthrosis (50).

No significant association between type of diet (such as home-prepared or commercial) and elbow and hip diseases was found; however high fat intake was positively associated with hip and elbow disease (50). Non-restricted feeding during growth and development has also been identified as a risk for developing both hip dysplasia and secondary hip osteoarthritis potentially a result of increased mechanical load in weight bearing joints (42). Furthermore, leptin has been found to be associated with osteoarthritis (81) and is found in higher levels in dogs that are overweight or obese (82), providing a possible alternative mechanism for osteoarthritis development. In studies conducted on paired littermates, one of which was on a control diet and the other on a restricted diet (25% less food than the control), dogs in the control group had an increased body weight and significantly increased development of osteoarthritis, which was also more severe. The onset of osteoarthritis was significantly delayed in the group with restricted intake (83). Therefore, as a modifiable risk factor, this provides evidence that appropriate feeding in order to maintain a lean body condition and therefore improved phenotype should be sustained throughout the dog's life to reduce the risk of joint disease (59).

#### Sex and Neuter Status

Neutered individuals were significantly more likely to have a joint disease compared to entire individuals in all studies that explored neutering as a risk factor, and therefore robust further study is needed to understand the possible relationship behind this. Associations between neutering and weight gain that have previously been highlighted in the literature (84) could at least in part explain the apparent increased risk of osteoarthritis development in neutered dogs also identified in this review [(16, 20, 25, 26, 34, 52, 85); Table 3]. Additionally, the impact of neuter status may be due to changes in gonadal hormones, higher levels of which can indirectly protect the joints and affect growth rates and development (52). It should be noted that for many of these studies the results may be heavily confounded by factors such as age. With neutered dogs most likely to be older on average than entire dogs, they may be at increased risk of development as a result of age rather than neuter status itself (86, 87).

Sex is also discussed as a risk factor in many studies, all reporting an increased risk associated with either being male or female, with some conflicting findings for individual disorders, likely a result of confounding by other factors not taken into consideration. This highlights the need for more comprehensive study of sex as a risk factor, and potential confounding factors and interactions between other risk factors. For example, differences in findings between sexes could also largely be a result of interactions of other confounding factors, such as body size and weight, neuter status, and hormone differences.

#### Age

There is no way to clearly determine when osteoarthritis or other joint conditions first developed, or when a predisposing disease process has or hasn't progressed into osteoarthritis, making age as a risk factor problematic and laden with assumptions. Many studies discuss aging as a potential risk factor, suggesting joint deterioration occurs increasingly with age and therefore suggesting older age as a risk factor for CCL and osteoarthritis. However, there is conflict in some papers' findings for dysplasia where increased risk was found in both younger and older dogs (Table 3). Again, many of these studies neglect to examine other variable interactions that could be involved in this progression deterioration. This conflict may further be a result of the reporting of chronic disease such as joint disorders, with a mix of reporting between prevalence or incidence across studies, and differences in terminologies for disease stage used across the studies. The incidence (new cases) may not be higher in older dogs but the prevalence (all cases within the population) would be expected to be higher in older dogs. Furthermore, although osteoarthritis may begin at any age, it may not be until it is clinically fulminant and reaches a more advanced stage that it is recognized as such. This is of particular concern in papers assessing primary care data (9). Therefore, findings related to age should be interpreted with caution, and methodological approach should be accounted for when assessing reliability of these findings. Longitudinal studies are warranted to explore the relationship between age and disease development more thoroughly.

#### Other Factors

Other notable risk factors reported by the literature include month of birth and early life factors such exercise levels and type. The link between month of birth and disease development is likely linked to exposure to differing exercise regimes when young. Those born in months that offer more favorable weather for exercise opportunities had increased risk of joint disease development. This is further supported through findings that identify exercise levels and types (such as chasing balls/toys and regularly playing with other dogs), throughout life but particularly when young, are risk factors for joint disease development, due to over-use of and damage to (developing) joints (43, 46, 47, 50, 53, 54, 71).

### Limitations of Evaluating Risk Factors for Canine Joint Disease

With conflicting findings such as age and inconclusive findings such as neutering, the limitations of this field of research in general, as well as the differences in aims and methodological approaches by the studies included in this review should be taken into consideration. Studies that investigate incidence (i.e., new cases of disease) are more likely to give more accurate data regarding age than prevalence studies, where age at diagnosis may be less obvious or available (14). Differences between study populations can also complicate comparisons across studies and result in inconsistencies. Referral dog populations (7) are not comparable to general populations as they are a sub-selection from this population, with the referral process potentially introducing selection bias which may lead to exaggerated findings. In studies that use primary-care veterinary data (9, 16, 75), diagnosis of joint diseases can vary greatly between individual veterinarians and veterinary practices. Furthermore, for some clinicians, clinical examination is enough, however others may require advanced imaging to make a diagnosis which can influence timing of diagnosis and therefore reported risk factors may be vastly different amongst studies. There may also be differences in terminology reported within studies causing further limitations, for example what one may call a hip dysplasia case, in reality may well already be clinical hip osteoarthritis, and reported as such by another. The time span across the literature included within this review is very large (1972–2018) and therefore changes for example in breed popularity and breed standards, research methodologies, clinical diagnostics and management, and even core veterinary knowledge over time may result in differences in findings. Finally, attention should be drawn to the number of studies on particular diseases, as well as particular risk factors. Although the corpus of 62 papers identified through the systematic evaluation process includes numerous joint diseases and conditions, the literature is fairly sparse for individual conditions. This relatively low number of papers reflects the need for further research in to risk factors for joint disease. The most frequently reported disease was hip dysplasia (32% of papers) and most frequently reported risk factor was genetics (34% of papers). Whilst this seemingly may imply that hip dysplasia is a high priority in veterinary medicine, and that genetics is the most influential or important risk factor for joint diseases, this could be simply resultant of research bias, and is more reflective of data availability and ease of access to pre-existing data. Further study into joint disease severity and prioritization, as well as risk factor weighting is warranted in order to quantify the influence of risk factors on disease.

Due to the diverse methodologies of epidemiological studies, no exclusions of literature were made based on study design, in order to include all papers that reported an increased risk of disease. As such, the database of papers included within this review is heterogeneous, and therefore it is unsurprising there are conflicting findings between studies, making comparisons limited and conclusions difficult to make. Furthermore, the majority of the studies (77%; 48 out of 62) in the existing literature are retrospective in design (see Table 3 for further study detail). As such, they are able to identify risk factors associated with development of osteoarthritis and joint diseases but are fundamentally unable to show causality. An understanding of causality is needed to move toward the development of effective control strategies. The strongest evidence for causality would therefore come from prospective longitudinal cohort studies (methodology adopted by only 23% of papers in this review). However, they also need appropriately calculated sample sizes to robustly identify and quantify risk factors across the lifetime of a dog, taking into account as many confounding factors as possible.

In order to return as many papers possible in the search output, only the Boolean operators “AND” and “OR” were used within search terms and these were searched for in all fields of papers (title, abstract, and full text). No terms were included as “NOT” so as to avoid inadvertently excluding possible references. However, whilst every effort was made to capture all current published papers on the topic of risk factors, it should be noted that some papers may still have been missed using this strategy, for example in the instance where they are not available on the searched databases. Furthermore, only results that focus specifically on canine osteoarthritis were included within this review, and therefore there may be risk factors identified in other canids and species which are not considered within this review. It should also be noted that misinterpretation of papers and reported data included in this review is always a possibility, along with human error in the systematic search, which may result in some literature being missed. As mentioned, limiting the inclusion criteria to articles published in peer-reviewed journals may have led to some level of unavoidable misrepresentation due to publication bias, however the limited availability and reliability of unpublished or non-peer reviewed gray literature makes this exceptionally hard to include. The QE scale used in this paper to evaluate reporting quality was adapted from a pre-existing scale to suit the heterogenous styles of literature, and therefore other factors may also influence the overall quality of these studies. The scores given in this paper are not a final grade but allow for comparison across the studies, and indications for key areas that studies lack in their reporting. In this scale, every point of the evaluation was equally weighted. In reality, certain points may be a greater indicator of quality than others, however, with an absence of evidence to support what this weighting should be, it was most appropriate to attribute equal weighting to all criteria. The evaluation of the papers is at least partly subjective and as such, inter-individual evaluations may differ. Whilst the percentage agreement score was high between the three assessors on this paper, such evaluations cannot be considered truly independent as the assessors were part of the same research team and are perhaps likely to share similar views on relevance.

### Reporting Quality and Future Studies

Reporting quality among the final corpus of 62 papers ranged from medium (5–7) to high (8, 9, 39) (Table 3), however, there is likely to be publication bias here as one of the requirements of this review is that included papers must have been peer-reviewed. It is therefore unsurprising that the papers are at least of medium quality (5/10). However, looking forward it is important that papers reporting quality and methodological design are of high quality in order to ensure reliability and validity of results and repeatability of studies. The most frequently occurring reasons for papers scoring below QE-H were, (i) that the research question was not clear, (ii) methods were not described fully, such that replication would be difficult, and (iii) potential bias such as sample selection. With these reasons in mind, in order to improve the reporting quality of future studies, it is recommended that a clear research question/hypothesis should be created prior to investigation and also reported in within the paper in a clear and concise manner. Subsequent methodology that appropriately samples the population and answers the research question yielding high quality and valid results is also needed, and sufficient detail should be provided regarded methodology within paper manuscripts. As discussed above, in the instance of risk factor analysis, longitudinal studies that can demonstrate causality would be of benefit to strengthen the current evidence base, and make future study comparisons more robust allowing more reliable conclusions to be drawn from the existing literature. Finally, with regards to reporting quality, only a small number of papers within this review reported sample size calculations (8%). Researchers should ensure these are both conducted and reported within future papers in order to form an appropriate sample population within their study, so that their results and findings that can be extrapolated confidently to the population of interest.

Due to the relatively small number of papers for a common condition in veterinary medicine, further studies are necessary in order to support the current papers' conclusions and extend the current evidence base. Research focus is particularly warranted where inconsistencies and conflicting outcomes have been found across the published studies. Specifically, a deeper understanding of the known risk factors contributing to joint diseases and identification of any as yet unreported factors, as well as the development of genetic screening tests, mapping of significant gene regions, and identifying gene functions would be particularly timely. Prevalence and incidence data of osteoarthritis resulting from predisposing conditions, as well as individual disease prevalence and incidence is currently lacking. There is also a lack of understanding of the nature of the interactions between known and potential risk factors not reported in the published literature. Different risks need to be further explored in order to determine their relative effect on disease development and severity, for example obesity vs. age, and understand their interactions. Finally, further exploration into early detection and diagnosis is needed in order to reduce the number of affected individuals that are bred from, and subsequently develop osteoarthritis.

## Conclusion

Here, a summary of published literature investigating risk factors for osteoarthritis and its predisposing conditions is presented. Six key risk factors were identified in the published literature, which were a mix of both modifiable and non-modifiable factors. Frequent reference to genetics is made in current literature highlighting a strong relationship between joint disease and certain genes related to growth and musculoskeletal development, as well as breed and conformational predispositions, highlighting “at risk” individuals. Identifying these individuals may allow for earlier diagnosis and management, and allow implementation of genetic and conformational screening programs to reduce inheritance into subsequent litters. Increasing body weight/condition was also found to have an association with joint disease, most likely due to the increased load on joints. Some identified risk factors such as age and neuter status warrant further investigation to understand more fully their relationship with joint disease, taking into account potentially confounding variables, particularly as there are other health and welfare benefits associated with aspects such as neutering. Other lifestyle risk factors are more easily managed and modifiable, such as the dog being overweight, and therefore preventative methods can be actioned directly. Osteoarthritis continues to be highly prevalent within the dog population, with substantial implications for quality of life and welfare. Understanding the key risk factors for the development of osteoarthritis and conditions that predispose it, is the first step to identifying means of controlling and ultimately reducing it within the population through preventative methods and control strategies. This study highlights these factors, as well as current literature gaps where further high-quality research is warranted.

## Data Availability Statement

The original contributions presented in the study are included in the article, further inquiries can be directed to the corresponding author.

## Author Contributions

KA conducted the systematic review, analyzed the outputs, and wrote and edited the paper. LC was responsible for study design and setting out the systematic review protocol, supported the analysis of outputs, and edited the paper. HZ, DO'N, and RM supported the study design and analysis of outputs, and edited the paper.

## Conflict of Interest

The authors declare that the research was conducted in the absence of any commercial or financial relationships that could be construed as a potential conflict of interest.
